# Supplementary material for: Genome-wide association study to identify SNPs and candidate genes associated with body size traits in donkeys
Source: Front Genet. 2023 Feb 28;14:1112377. doi: 10.3389/fgene.2023.1112377 (PMC10011486; doi:10.3389/fgene.2023.1112377)
Supplement: Supplementary file 1 [file DataSheet1.PDF]

Supplementary material

Genome-wide association study to identify SNPs and candidate genes associated with body size traits in donkeys

Shuang Song[1†], Shiwei Wang[1†], Nan Li[1], Siyu Chang[1], Shizhen Dai[1], Yajun Guo[1], Xuan Wu[1], Yuanweilu Cheng[1], Shenming Zeng[1*]

[1] Key Laboratory of Animal Genetics, Breeding, and Reproduction of the Ministry of Agriculture, College of Animal Science and Technology, China Agricultural University, Beijing, 100193, China

[†] These authors contributed equally to this work.

*Corresponding author: zengsm@cau.edu.cn

The additional file 1 includes:

Figure S1

The title and legend for Table S1 to S5

The additional file 2 includes:

Table S1 to S5

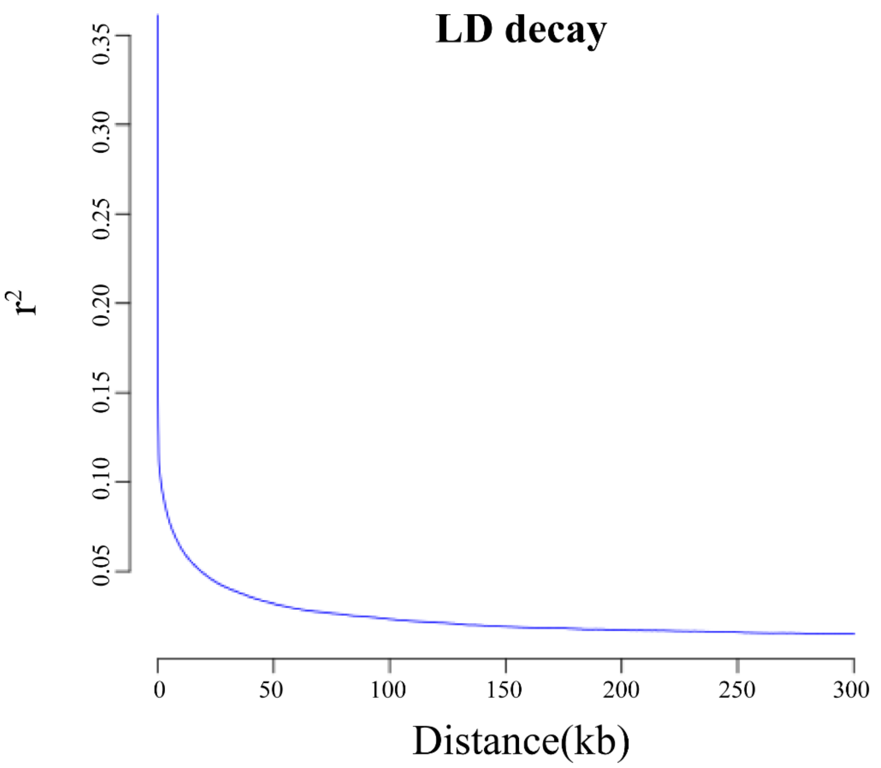

Figure S1. Linkage disequilibrium analysis.

Table S1. Raw measurement data of the four body size traits.

Table S2. Raw sequencing data statistics and quality assessment. Read Num.: total number of reads; Total Bases (bp): total number of bases. N(%): percentage of fuzzy bases; GC(%): GC content; Q20(%): percentage of base recognition accuracy above 99%; Q30(%): percentage of base recognition accuracy above 99.9%.

Table S3. Sequence alignment result statistics. Total reads: total number of sequences; mapping rate (%): the proportion of the number of sequences that could be aligned to the reference genome in the total number of sequences; Dup. nums: number of repeated sequence reads; Dup. rate: percentage of the number of repetitive reads to the total number of reads.

Table S4. Sequencing depth and coverage statistics. Avg. depth (×): average sequencing depth, the total number of bases aligned to the reference genome divided by the genome size; coverage≥1(%): the percentage of genes covered by at least one sequence in the reference genome; coverage≥4 (%): the percentage of genes covered by at least four sequences in the reference genome; coverage≥10 (%): the percentage of genes covered by at least 10 sequences in the reference genome.

Table S5. Candidate genes and their GO and KEGG analysis results.
